# Analyse biochimique multi-paramétrique révélant une augmentation de l'homocystéinémie et du NT-ProBNP chez les patients hypertendus à Bamako (Mali)

**DOI:** 10.11604/pamj.2020.35.10.18821

**Published:** 2020-01-13

**Authors:** Yaya Goïta, Juan Manuel Chao de la Barca, Asmaou Keita, Mamadou Bocary Diarra, Klétigui Casimir Dembélé, Boubacar Sidiki Ibrahim Dramé, Yaya Kassogué, Mahamadou Diakité, Françoise Joubaud, Marie-Christine Denis, Chadi Homedan, Delphine Mirebeau-Prunier, Pascal Reynier, Bakary Mamadou Cissé, Gilles Simard

**Affiliations:** 1 Faculté de Pharmacie, Université des Sciences, des Techniques et des Technologies de Bamako, Mali; 2 Centre Hospitalier Universitaire Mère-Enfant (CHUME), Service de Cardiologie & Laboratoire d'Analyses Médicales, Bamako, Mali; 3 Departement de Biochimie et Génétique, Centre Hospitalier Universitaire, Angers, France; 4 Laboratoire de Biologie médicale, Centre Hospitalier Universitaire Hôpital du Mali, Bamako, Mali

**Keywords:** Hyperhomocystéinémie, NT-ProBNP, hypertension artérielle, Homocysteine, Nt-ProBNP, Arterial Hypertension

## Abstract

**Introduction:**

L'hypertension artérielle est un problème majeur de santé publique en Afrique subsaharienne par sa fréquence élevée et le risque cardiovasculaire qu'elle entraine. L'objectif de cette étude était d'évaluer la prévalence des facteurs de risques cliniques et biologiques de l'hypertension artérielle à Bamako (Mali).

**Méthodes:**

Il s'agit d'une étude cas-témoin, stratifiée en fonction du sexe, portant sur 72 participants dont 36 hypertendus et 36 contrôles. Vingt-deux paramètres biochimiques plasmatiques ont été mesurés et analysés par des tests univariés et multivariés.

**Résultats:**

Une hyperhomocystéinémie a été retrouvée chez 55,6% des femmes (p = 0,03) et 100% des hommes (p = 0,007) hypertendus. Le N-terminal pro B-type natriuretic peptide (NT-Pro-BNP) était également augmenté chez 16,7% des femmes (VIP > 1 dans le modèle multivarié) et des hommes hypertendus (p = 0,00006). Un bon modèle multivarié prédictif (OPLS-DA) a uniquement été obtenu chez les femmes hypertendues, avec un Q^2^cum = 0,73, attestant ainsi d'un important dimorphisme sexuel associé à l'hypertension artérielle. Ce modèle impliquait huit paramètres dont la concentration plasmatique était modifiée (homocystéine, NT-Pro-BNP, potassium, urée, glycémie, sodium, chlore et protéines totales).

**Conclusion:**

Nous avons noté une association significative entre l'hyperhomocystéinémie et l'hypertension artérielle. Par conséquent, le dosage de l'homocystéine associé à une bonne prise en charge diminuerait le risque cardiovasculaire tout en améliorant la qualité de vie des patients hypertendus.

## Abstract (en)

**Introduction:**

Arterial hypertension is a major public health problem in sub-Saharan Africa due to its high frequency and to the cardiovascular risk that it entails. The purpose of this study was to assess the prevalence of clinical and biological risk factors of hypertension in Bamako (Mali).

**Methods:**

We conducted a case-control study, stratified in function of the sex, of 72 participants including 36 patients with hypertension and 36 controls. Twenty-two plasma biochemical parameters have been measured and analyzed using univariate and multivariate tests.

**Results:**

Hyperhomocysteinemia was found in 55.6% of women (p = 0.03) and 100% of men (p = 0.007) with hypertension. High NT-proBNP was also found in 16.7% of women (VIP > 1 in multivariate model) and of men with hypertension (p = 0.00006). A good multivariate predictive model (OPLS-DA) was only obtained in women with high blood pressure, with Q^2^cum = 0.73, attesting severe sexual dimorphism associated with arterial hypertension. This model involved eight parameters whose plasma concentration was modified (homocysteine, NT-proBNP, potassium, urea, blood glucose, sodium, chlorine and total proteins).

**Conclusion:**

We registered a significant association between hyperhomocysteinemia and arterial hypertension. Therefore, the assay of homocysteine associated with good management would decrease the risk of cardiovascular diseases while improving the quality of life of hypertensive patients.

## Introduction

L'hypertension artérielle (HTA) est définie par une pression artérielle systolique (PAS) supérieure à 140 mmHg et/ou une pression artérielle diastolique (PAD) supérieure à 90 mmHg de manière répétée, en position allongée après 10 minutes de repos. Des stades 1 (140 < PAS < 159 mmHg et/ou 90 < PAD < 99 mmHg), 2 (PAS ≥160 mmHg et/ou PAD ≥100 mmHg) et 3 (PAS ≥ 180 mmHg et/ou PAD ≥ 110 mmHg) ont été définis selon le *Joint National Committee* (JNC) [1]. L'HTA est un facteur chronique majeur de risque cardiovasculaire dont les causes sont multiples et souvent difficiles à mettre en évidence, comme c'est le cas pour l'HTA dite essentielle que l'on retrouve dans la grande majorité (~90%) des cas [2]. Elle concernerait 1,13 milliard d'individus dans le monde [3] et serait responsable de 7,6 millions de décès par an [4]. La prévalence de l'HTA augmente avec l'âge pour atteindre un plateau après 60 ans. Les principaux facteurs de risque de l'HTA essentielle sont l'âge, le poids, le diabète, le sexe masculin, la sédentarité, l'hérédité et la consommation excessive de sel ou d'alcool [5]. L'HTA du sujet noir africain est plus sévère et se développe plus précocement, avec un pourcentage plus élevé de complications que chez le sujet européen [6]. La prévalence de l'HTA en Afrique de l'ouest dans la population adulte est estimée à 40% [7] et elle est en forte augmentation dans l'Afrique sub-saharienne [3,6]. Des facteurs génétiques favorisant la rétention du sel et d'eau expliqueraient cette fréquence et cette gravité particulières de l'HTA du sujet africain [8]. Au Mali, l'HTA varie selon les études de 20,83% à 39,4% [9,10], sans influence apparente de l'origine ethnique [9]. Méconnue ou mal soignée, l'HTA peut causer de graves dommages au niveau des artères cérébrales, cardiaques et rénales, favorisant l'apparition de complications graves, telles que les accidents vasculaires cérébraux, l'infarctus du myocarde et les insuffisances cardiaque ou rénale. Ces complications aboutissent fréquemment à des décès ou à des invalidités précoces [11]. Au Mali, la mortalité liée au retard diagnostique et à ses complications est très élevée, variant de 16,6% à 32,5% selon les études [12]. L'objectif de notre étude est de mieux définir dans la population malienne, les facteurs de risques cardiovasculaires biologiques et cliniques associés à l'HTA, afin d'améliorer la prise en charge et de réduire les complications cardiovasculaires.

## Méthodes

**Aspects éthiques**: l'étude a été menée conformément aux normes de l'éthique énoncées dans la Déclaration d'Helsinki (1983) et approuvée par le comité d'éthique de la Faculté de Médecine, de Pharmacie et d'Odonto-Stomatologie (FMPOS) de l'Université des Sciences, des Techniques et des Technologies de Bamako (l'USTTB) suivant le numéro 2016/05/CE/FMPOS du 18 janvier 2016. L'inclusion dans l'étude des participants était conditionnée à la signature de la fiche de consentement éclairée, libre et volontaire déposée au niveau de la commission d'éthique du Mali.

**Type d'étude et participants**: c'était une étude cas-témoins qui a inclus 72 individus de plus de 30 ans, dont 36 hypertendus (18 hommes et 18 femmes) et 36 témoins non hypertendus (18 hommes et 18 femmes). Tous les patients hypertendus inclus dans l'étude étaient suivis en ambulatoire ou hospitalisés dans le Service de Cardiologie du Centre Hospitalier Universitaire Mère-Enfant (CHUME) le Luxembourg de Bamako et avaient une pression artérielle supérieure à 140/90 mmHg. Les sujets témoins ont été sélectionnés parmi les individus accompagnant d'autres patients au CHUME. Les critères d'inclusion étaient l'absence d'hypertension artérielle vérifiée (<140/80 mmHg) et de facteur de risques cardiovasculaire. Le recrutement des participants a été réalisé d'octobre 2017 à mars 2018.

**Collecte des échantillons**: les prélèvements sanguins ont été réalisés le matin après 12 heures de jeûne dans des tubes héparinés dans le laboratoire d'analyses médicales du CHUME et centrifugés à +4°C immédiatement pendant 10 minutes à 3000g et avant récupération du plasma. Les plasmas ont été aliquotés en 1ml et conservé à -80°C avant leur transport dans la glace au laboratoire de biochimie et biologie moléculaire du CHU d'Angers, France pour être analysés.

**Paramètres explorés lors de l'étude**: les données sociodémographiques (âge, sexe), la tension artérielle (systolique/diastolique), les antécédents médicaux (diabète, insuffisance rénale, insuffisance cardiaque), le tabagisme, les traitements (les antihypertenseurs) des patients hypertendus et contrôles ont été recueillis. Les paramètres biologiques dosés étaient: l'homocystéinémie, NT-Pro-BNP, CRP ultrasensible (CRPus), la glycémie, ionogramme sanguin (sodium, potassium et chlore), protéines totales, transaminases, gamma GT, cholestérol total, HDL et LDL cholestérol, ApoA, ApoB, Lp(a), urée, créatinine et la vitamine B12. Le diabète a été déclaré et confirmé par le dosage de la glycémie à jeun > 1,5g/L (8,3mmol/L), l'hyperhomocystéinémie par une valeur d'homocystéine > 18μmol/L (normale: 5 à 15μmol/L), l'insuffisance cardiaque par le dosage du NT-Pro-BNP (valeurs d'inclusion > 450 ng/L pour un âge < 50 ans ; > 900 ng/L pour un âge compris entre 50-75 ans) [13]. L'insuffisance rénale a été déterminée après calcul du débit de la filtration glomérulaire (eDF) à partir de l'Equation MDRD (Modification of diet in renal disease), eDFG = 175 x (Scr x 0,0113)-1,154 x âge-0,203 x 0,742 (si femme) x 1,212 (noir africain), (Créatinine sérique (Scr) = μmol/L et âge en année) [14]. Les hypoprotéinémies étaient basées sur une protéinémie < 65 g/L, les hyperprotéinémies sur un dosage > 80 g/L. Les hyponatrémies étaient basées sur un sodium plasmatique < 135 mmol/L, les hypernatrémies sur un sodium > 145 mmol/L. Les hypokaliémies étaient basées sur une concentration de potassium < 3,5 mmol/L, les hyperkaliémies sur une concentration > 4,5 mmol/L. Les hypochlorémies correspondaient à un taux de chlore plasmatique < 95mmol/L, les hyperchlorémies à une concentration > 105mmol/L, le syndrome-inflammatoire était déterminé par une valeur supérieure à 6mg/L de la mesure de la CRPus [15]. La surcharge pondérale a été classée après calcul de l'indice de masse corporelle (IMC: kg/m^2^). Les participants ont été classés en non obèses (IMC = 16 à 24,9) ou obèses (IMC = 25 à 39,9) [16].

**Analyse de l'homocystéine par spectrométrie de masse en tandem**: la préparation des échantillons et les analyses ont été réalisées en suivant la procédure de la méthode de Mark J *et al*. 1999 [17]. L'homocystéine et l'homocystéine D8 ont été achetées respectivement auprès de Sigma et Cambridge *Isotope Laboratory*. Une fois réduite, l'homocystéine-D8 donne l'homocystéine-d4 (Hcy-d4) au double de la concentration initiale. La réduction a été réalisée par addition de dithiothréitol (DTT). L'analyse nécessitait un prétraitement de l'échantillon biologique (plasma) par simple précipitation des protéines à l'éthanol, suivi d'une centrifugation. Cinq microlitres du surnageant additionnés à une quantité fixe de d'Hcy-d4 ont alors été injectés sur une colonne C 18 en phase inverse (Supelcosil^™^, 3 μm, LxI.D.3,3 cm x 4,6mm. couplée à un spectromètre de masse quadripolaire API 3000 Applied Biosystems^®^ équipé d'une source d'ionisation electrospray (TurboR IonSpray). Les périphériques comprenaient une pompe Agilent 1100 Séries et un passeur automatique d'échantillons. La phase mobile était composée de 95% d'acétonitrile, 0,1% d'acide formique et 5% d'eau et la quantité d'échantillon injectée était de 5μL. Dans le mode de surveillance de réactions multiples (MRM), l'instrument a été optimisé automatiquement par l'algorithme intégré pour suivre les transitions de 136,1 à 90m/z et de 140,1 à 94m/z pour respectivement tHcy et Hcy-d4. Les données brutes acquises ont ensuite été traitées en utilisant le logiciel Analyst Sciex, pour quantifier l'homocystéine des échantillons à partir d'une droite d'étalonnage réalisée en exprimant, à partir de concentrations connues et croissantes en homocystéine, le ratio tHcy/Hcy-d4.

**L'analyse des paramètres biochimiques**: le bilan biochimique a été réalisé sur les automates ADVIA *ChemistryXPT* et *CentaurXPT* (Siemens). Les kits de dosages, les calibrateurs et les contrôles ont été achetés auprès des laboratoires Siemens et Bio-Rad. Les sérums de contrôles utilisés sur ADVIA CentaurXPT étaient de: *Liquichek^™^ cardiac Markers plus Control LT* (lot 23650), utilisé pour le contrôle de qualité de l'analyte NT-ProBNP et *Liquichek ^™^ immunoassay Plus Control* (lot 40920), utilisé pour le contrôle de qualité de la vitamine B12 ; sur Advia *Chemistry XPT: Liquid Assayed Multiqual* (lot 45770) utilisé pour le contrôle des analytes suivants: le sodium, l'ion chlorure, le potassium, le cholestérol HDL, le cholestérol LDL, le cholestérol total, les triglycérides, la créatinine, la gamma glutamyl transférase (GGT), le glucose, les protéines totales et le contrôle *Immunology* (lot 66370) pour la CRP_us_.

**Analyses statistiques univariées**: le test non paramétrique de Mann-Whitney-Wilcoxon a été utilisé pour comparer les médianes des variables quantitatives tandis que les tests de χ^2^ ou le test exact de Fisher, selon les effectifs, ont été utilisés pour les variables qualitatives. Les différences ont été considérées comme significatives si la probabilité (p) de la différence observée sous l'hypothèse nulle était inférieure ou égale à 0,05 (p≤ 0,05).

**Analyses statistiques multivariées**: l'analyse multivariée utilisée est basée sur les méthodes de projection de type non supervisée (analyse en composantes principales ou PCA) et supervisée (moindres carrés partiels associés à l'analyse discriminante ou OPLS-DA). La PCA permet la détection du groupe d'échantillons similaires et des individus ayant des valeurs aberrantes (*outliers*). OPLS-DA a ensuite été appliqué pour maximiser les variations entre les groupes hypertendus et témoins, afin de déterminer l'existence d'une variable latente discriminant les groupes comparés ainsi que les paramètres contribuant à cette variation, le cas échéant. Les valeurs de VIP (*Variable Important for the Projection*) résument l'importance de chaque variable pour le modèle OPLS-DA tandis que les *loadings* prennent en compte le poids de chaque paramètre biologique dans la variable latente. La qualité du modèle OPLS-DA a été validée par deux paramètres, le coefficient de détermination (R^2^) et par la valeur du coefficient de détermination cumulé sur toutes les variables latentes du modèle et obtenu après validation croisée ou Q^2^cumulée (Q^2^cum). Un seuil de 0,5 a été utilisé pour déterminer si un modèle OPLS-DA pouvait être estimé comme ayant une bonne capacité prédictive (Q^2^cum ≥ 0,5) ou médiocre (Q^2^cum< 0,5). Cette analyse multivariée a été menée en utilisant le logiciel SIMCA-P v.14.1 (Umetrics, Umeà, Suède).

## Résultats

**Caractéristiques socio-démographiques, antécédents, mode de vie et traitements**: sur les 72 participants, les âges médians étaient respectivement de 40,0 ± 7,1 ans (extrêmes de 36 à 58 ans) et 47,5 ± 6,2 (36-55 ans) chez les hommes hypertendus (n = 18) et chez les contrôles (n =18), avec une différence non significative entre les deux groupes (p = 0,073). Ils étaient de 40,5 ± 7,3 ans (35-60 ans) et 46,5 ± 4,4 ans (34-51 ans) chez les femmes hypertendues (n = 18) et les contrôles (n = 18), avec une différence non significative entre les deux groupes (p = 0,067). La médiane des tensions artérielles (Systolique/Diastolique) était de 170/110 mmHg pour les hommes hypertendus et de 120/80 mmHg pour les hommes contrôles, avec une différence significative entre les deux groupes (p = 0,0001). Les médianes étaient de 170/110 mmHg pour les femmes hypertendues et de 120/80 mmHg pour les femmes contrôles, avec une différence significative entre les deux groupes (p = 0,0001). Selon la classification de l'HTA définie par le JNC, 66,7% des sujets hypertendus étaient classés en stade 2 (>160/100 mmHg) et 22,2% en stade 3 (>180/110 mmHg). Sur la base de l'interrogatoire, le régime alimentaire hyposodé était suivi par 77,77% des hommes et 66,7% des femmes. Tous les patients hypertendus de sexe féminin étaient sous traitement antihypertenseur (beta-bloquants et diurétiques thiazidiques) contre 66,66% des hommes hypertendus. La seule différence significative en termes de traitement était la prise de préparations à base de plantes à propriétés anti-hypertensives qui a été indiquée par 22,22% des hommes et aucune femme (p = 0.027). Parmi les patients hypertendus, 72,2% des hommes et 94,4% des femmes étaient en surpoids ou obèses. Environ soixante-douze pourcent (72,2%) des hommes et 61,1% des femmes hypertendus avaient une activité physique faible ou nulle. L'incidence du diabète dans notre population d'hypertendus était plus élevée chez les femmes (27,8% pour les femmes hypertendues et 16,67% pour les hommes hypertendus, p = 0.019). De plus, nous n'avons pas observé de différence quant à la prévalence de l'insuffisance rénale, 5,6% des hommes et des femmes présentant une insuffisance rénale modérée.

**Figure 1:**
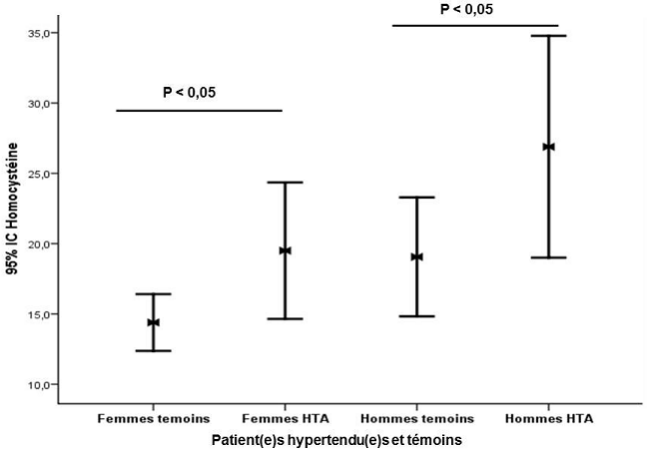
Représentation graphique des valeurs de l'homocystéine en fonction des sujets témoins et HTA

**Paramètres biologiques**: l'analyse univariée (Tableau 1) a été utilisée pour explorer les paramètres biochimiques. Ainsi, l'hyperhomocystéinémie (p = 0,007 chez les hommes et p = 0,03 chez les femmes (Figure 1) et l'hypochlorémie (p = 0,04 chez les hommes et p = 0,000006 chez les femmes) étaient les seuls paramètres significativement modifiés à la fois chez les hommes et les femmes hypertendus comparées à leurs contrôles respectifs. Les paramètres suivants étaient significativement modifiés uniquement chez les hommes hypertendus comparés aux contrôles: NT-ProBNP (p = 0,00006) et CRPus augmentées (p = 0,04). Les paramètres suivants étaient significativement modifiés uniquement chez les femmes hypertendues comparées aux contrôles: glycémie augmentée (p = 0,019), sodium abaissé (p = 0,000003), potassium augmenté (p = 0,0003) et la protéinémie abaissée (p = 0,003), GGT abaissée (p = 0 ,045).

**Tableau 1:** Déterminants cliniques et biologiques des sujets témoins et hypertendus

Paramètres (médianes)	Hommes (n = 18)			Femmes (n = 18)		
	HTA	Non HTA	p	HTA	Non HTA	p
Age (ans)	47,5(36-55)	40(36-58)	0,073	46,5((34-51)	40,5(35-60)	0,067
PAS (mmHg)	16,5(15-18)	120(11-13)	0,0001*	17,0(16-18)	12,0(10-13)	0,0001*
PAD (mmHg)	11(9-12)	80(7-8)		11(10-12)	8,0(7-8)	
Hyposalé %	14(77,8)	0	0,0001*	12(66,7)	0	0,0001*
Non salé %	4(22,2)	0		6(33,3)	0	
IMC/Kg/m²	26(16-34)	22(18-35)	0,038*	29(24-35)	21,5(17-24)	0,0001*
Gly (mmol/L)	4,3(2,0-9,0)	4,8(4,0-6,0)	0,735	9,6 (3,0-12,6)	4,4 (3,6-6,0)	0,019*
Créat (μmol/L)	84(62-425)	85(60-110)	0,198	56(39-1091)	53(38-78)	0,223
Urée (mmol/L)	3,8(1-27,9)	3(2-4,3)	0,08	3,4(2-24)	3(2,0-3,6)	0,084
eDF (mL/mn)	108(17-155)	110(85-156)	0,277	129(4-195)	142(87-205)	0,325
Hcyst (μmol/L)	20,5(16-78)	18(12-50)	0,007*	16(11-52)	13,5(9-24)	0,03*
NTPBNP (ng/L)	117,5(35-8522)	35(35-116)	0,00006*	63(35-5850)	52(35-276)	0,134
Na^+^ (mmol/L)	138(125-145)	140(136-142)	0,056	135,5(131-141)	141,5(138-145)	0,000003*
Cl^-^(mmol/L)	102(81-108)	105(100-108)	0,04*	101,5(98-107)	107(102-111)	0,000006*
K^+^(mmol/L)	3,6(2,1-5,8)	3,7(3,1-4,2)	0,762	4,35(3,8-6,0)	3,9(3,0-5,4)	0,0003*
Prot (mg/L)	72,5(55-86)	73,5(67-80)	0,884	71,5(62-81)	75(69-91)	0,003*
CRPus( mgL)	2,9(0,2-15,1)	0,9(0,2-6,2)	0,04*	4,4(0,2-23,9)	0,8(0,2-9,1)	0,168
Chol (mmol/L)	4,9(2,4-7,3)	4,2(2,9-5,6)	0,722	4,6(2,8-7,4)	4,1(3,1-6,3)	0,417
HDL-chol (mmo/L)	1,1(0,5-1,6)	1(0,55-1,47)	0,329	1,10(0,8-2,0)	1,15(0,90-1,40)	0,944
LDL-chol (mmol/L)	2,5(1,3-5,0)	2,65(1,5-3,6)	0,433	2,70(1,50-5,20)	3,10(1,60-4,70)	0,508
TG (mmol/L)	0,8(0,37-1,68)	0,91(0,45-3,56)	0,208	0,92(0,43-2,29)	0,85(0,33-1,51)	0,187
ApoA1 (g/L)	1,2(0,69-1,61)	1,18(0,91-1,30)	0,522	1,30(1,07-1,62)	1,22(0,64-1,46)	0,320
ApoB (g/L)	0,84(0,56-1,58)	0,78(0,45-1,09)	0,227	0,88(0,48-1,81)	0,75(0,48-1,35)	0,263
Lp(a) (g/L)	0,20(0,10-0,85)	0,20(0,10-0,8)	0,360	0,20(0,10-0,60)	0,20(0,10-0,5)	0,627
GGT (UI/L)	30(17-304)	30,5(14-77)	0,346	30(13-330)	31(7-38)	0,045*
ASAT (UI/L)	22(16--35)	22,5(13-54)	0,481	17,5(12-36)	22(15-32)	0,091
ALAT (UI/L) Vit B12	15(9-27) 476,5(135-815)	15(9-33) 417,5(239-833)	0,561 0 ,399	11(9-38) 487(268-903)	10,5(9-29) 505(183-770)	0,706 0,921

* significativement associé

PAS: Pression artérielle systolique, PAD: Pression artérielle diastolique, IMC : Indice de masse corporelle, Gly: glycémie, Créat: créatinine, eDF: Débit de la filtration glomérulaire , Hcyst : homocystéine, Na+: sodium, Cl: chlore, K+: potassium, Prot: protéine, CRPus: protéine C-réactive ultra-sensible, Chol: cholestérol, HDL-Chol: lipoprotéine de haute densité, LDL-Chol: lipoprotéine de faible densité, TG: triglycérides, ApoA1: apoliprotéine A1, ApoB: apoliprotéine B, Lp(a) lipoproteine a, GGT: gamma glutamyl-transférase, ASAT: aspartate amino-transférase, ALAT: aAlanine amino-transférase, Vit B12: vitamine B12.

**Figure 2:**
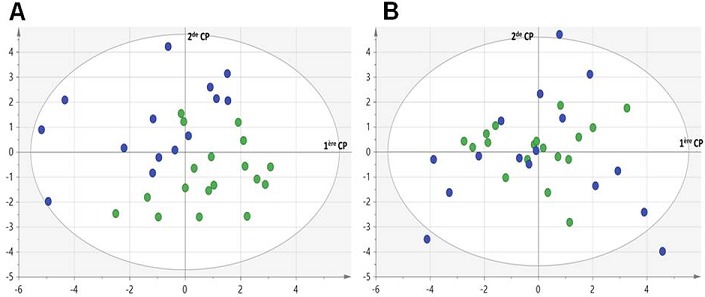
Premier plan principal de la PCA chez les femmes (A) et chez les hommes (B) après avoir éliminé les individus atypiques. On remarque une discrète séparation entre les femmes hypertendues (cercles bleus) et les femmes contrôles (cercles verts) dans le premier plan principal de la PCA. Chez les hommes aucune séparation entre les hypertendus (cercles bleus) et contrôles (cercles verts) n'est apparente. Les unités des composantes principales (CP) sont arbitraires

**Figure 3:**
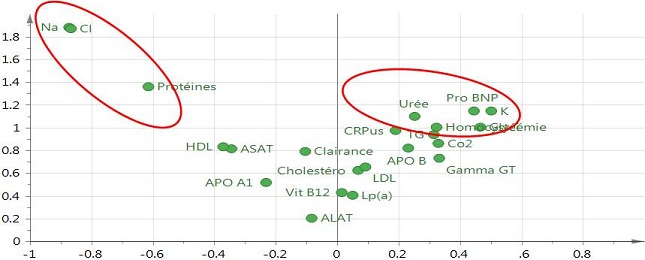
Loadings (abscisse) et VIP (ordonnée) du modèle OPLS-DA prédictif obtenu chez les femmes. Les paramètres avec une valeur de VIP≥1 sont considérés comme importants pour le modèle multivarié

**Analyse multivariée**: l'analyse des composantes principales nous a conduit à exclure 6 patients hypertendus considérés comme *outliers* dont 4 femmes et 2 hommes. Finalement, l'analyse multivariée a porté sur 32 femmes et 34 hommes. Chez les femmes, la PCA montrait une discrète séparation entre les femmes hypertendues et les contrôles (Figure 2), tandis que chez les hommes aucune séparation entre ces deux groupes n'était visible. L'analyse supervisée (OPLS-DA) a conduit à un modèle multivariée prédictif uniquement chez les femmes (*Q^2^cum* = 0,73) car chez les hommes hypertendus le Q^2^cum était inférieur au seuil de discrimination fixé à 0,5 (*Q^2^cum < 0,5*). Les meilleurs paramètres discriminants (avec des valeurs VIP > 1 et *loadings* élevées) contribuant à ce modèle comprenaient un sous-ensemble de 8 paramètres, avec pour cinq d'entre eux une concentration plasmatique relativement plus élevée chez les femmes hypertendues comparées aux contrôles (homocystéine, NT-Pro-BNP, potassium, urée, glycémie), tandis que pour les trois paramètres restant (sodium, chlore et protéines totales) la concentration plasmatique était relativement plus basse chez les femmes hypertendues comparées aux contrôles. L'ensemble des VIPs et *loadings* du modèle final OPLS-DA pour les femmes est représenté dans la Figure 3.

## Discussion

Nous avons mené une étude cas témoins sur l'hypertension artérielle, chez 36 patients et 36 contrôles stratifiés en fonction du sexe. La fréquence d'apparition de l'HTA croît avec l'âge jusqu'à 50 ans et ceci dans les deux sexes. Les âges médians de nos patients hypertendus étaient de 47,5 chez les hommes et 46,5 ans chez les femmes sans différence significative avec les groupes témoins. Ces chiffres sont comparables à ceux observés par Carrère *et al.* [18] dans une population guadeloupéenne d'origine africaine, avec des médianes de 46,6 ans pour les hommes et 47,2 ans pour les femmes hypertendus. Une étude menée au Mali [9] a rapporté que, chez les femmes l'HTA s'observe dans toutes les tranches d'âges mais plus de la moitié se rencontre au-delà de 40 ans, la distribution de l'HTA chez les hommes montrant que le tiers des malades avait plus de 40 ans. Dans notre étude, seulement 22,22% des sujets présentaient une HTA sévère. Ce pourcentage bien inférieur à celui rapporté dans la population urbaine de Bouaké [19] pourrait s'expliquer par l'âge moyen plus jeune de la population de notre étude. Parmi les facteurs de risque cliniques classiques, nous n'avons pas observé de prévalence accrue du tabagisme ou de l'alcoolisme chez nos patients hypertendus. La prévalence de l'insuffisance rénale constatée aux différents stades était aussi inférieure aux résultats précédemment observés au Mali [20] ou en Guinée [21]. En revanche, les sujets diabétiques, sédentaires, obèses ou insuffisants rénaux étaient beaucoup plus fréquents parmi les patients hypertendus alors qu'ils n'étaient pas ou peu retrouvés dans notre population témoin. L'incidence de l'HTA est plus élevée chez le patient obèse sédentaire et cette association morbide conduit à une augmentation de la morbi-mortalité cardiovasculaire [22]. Cette forte proportion de diabète et d'obésité associée à l'HTA dans notre étude est très supérieure à celle précédemment rapportée au Mali [20] ou dans d'autres populations de race noire [19,23]. Le fait que les patients de notre étude résident majoritairement à Bamako et dans ses environs explique sans doute cette différence. En effet, l'urbanisation croissante en Afrique s'accompagne de changements profonds de mode de vie, de l'alimentation, de la sédentarité qui tendent à favoriser le surpoids et l'insulino-résistance [24]. De manière surprenante, notre étude n'a pas observé une fréquence accrue des anomalies lipidiques chez ces hypertendus, en dépit de cette forte prévalence du diabète et de l'obésité. Par conséquent, nous n'avions pas retrouvé l'association récemment rapportée d'une diminution du cholestérol-HDL et de l'apolipoprotéine A1 chez les hypertendus présentant une hyperhomocystéinémie [25].

Nous avons observé une augmentation significative de l'homocystéine dans les deux sexes. Si l'association entre des taux élevés d'homocystéine et l'augmentation du risque de maladies coronariennes et du risque d'ischémie cérébrale est étudiée depuis de nombreuses années [26,27], le concept d'une plus forte prévalence de l'hyperhomocystéinémie chez les hypertendus dans certaines populations commence à émerger [28,29]. L'explication de cette association reste hypothétique. L'hyperhomocystéinémie par son action délétère sur la paroi des vaisseaux sanguins (stress oxydatif, inhibition de la croissance des cellules endothéliales,…) pourrait promouvoir ou accélérer le développement de l'hypertension. Quel qu'en soit le mécanisme, la présence de cette hyperhomocystéinémie potentialiserait le risque cardiovasculaire lié à l'hypertension artérielle et mériterait donc d'être prise en charge chez ces patients. Toute une série de facteurs génétiques, nutritionnels, ou liés au mode de vie (tabagisme) sont susceptibles d'entrainer une augmentation de la concentration plasmatique en homocystéine. Parmi les facteurs nutritionnels, des carences en vitamine B12 et en acide folique sont souvent rapportées dans les populations où la consommation de fruits et légumes frais est faible. En Afrique de l'Ouest (Bénin, Togo), les taux bas en folate ont été observés dans la population générale à la différence de la vitamine B12 dont les taux sont comparables voire supérieurs à ceux des pays occidentaux [30,31]. Nous n'avons malheureusement pas pu mesurer les folates. En revanche, nous n'avons pas observé de différence significative de concentration en vitamine B12 chez les sujets hypertendus, suggérant qu'une carence en folate pourrait être en partie responsable de l'hyperhomocystéinémie. Celle-ci gagnerait donc à être systématiquement recherché et corrigé chez les sujets hypertendus au Mali. Le NT-proBNP est le deuxième paramètre trouvé significativement différent chez les hommes hypertendus (p = 0,00006) en analyse univariée et chez les femmes hypertendues en analyse multivariée. Cette augmentation du NT-proBNP est le signe biologique d'une insuffisance cardiaque observée chez 16,7% des hommes et des femmes de cette étude [32,33]. Cette incidence est légèrement inférieure à celle rapportée précédemment (30%) dans la population malienne [20], mais bien supérieure à celle réalisée au Togo (5,5%) [23]. A côté de son rôle diagnostique, le NT-proBNP est aussi un marqueur prédictif du risque d'évènements cardiovasculaires graves, d'AVC ou de mort subite [34,35]. Cette capacité prédictive serait même supérieure à celle de la CRP ultrasensible [36]. De nombreuses études dont une réalisée au Togo [23] ont confirmé l'utilité de ce dosage [37,38] pour stratifier le risque cardiovasculaire. Il pourrait donc avoir sa place pour stratifier le risque cardiovasculaire des sujets hypertendus maliens.

La protéine C-Réactive ultrasensible (CRP*_us_*) n'a été trouvée significativement augmentée que chez les hommes hypertendus (p = 0,04) en analyse univariée. La dispersion des valeurs de CRPus est connue chez la femme en période de cycle menstruel [39]. Elle pourrait expliquer cette absence de significativité entre HTA et CRP*_us_* chez les femmes. L'inflammation pourrait être un élément reliant l'hypertension artérielle aux complications cardiovasculaires et elle pourrait être secondaire à l'hyperhomocystéinémie. Une étude réalisée sur le lien entre CRP*_us_* et HTA rapporte qu'en analyse univariée, la variabilité de la pression artérielle systolique de repos était la seule variable indépendante liée à la CRP*_us_*, la protéine C-réactive étant significativement (p = 0,034) associée à un risque accru d'hypertension artérielle [40]. Une étude réalisée aux Etats-Unis [41] indique qu'une inflammation systémique de faible grade et une hyperhomocystéinémie étaient associées à un risque élevé de coronaropathie sur 10 ans. La relation de causalité entre CRP*_us_* et HTA reste toutefois difficile à affirmer, une pression systolique élevée et/ou une variabilité de la pression systolique élevée pouvant provoquer des phénomènes inflammatoires vasculaires [42]. En plus des marqueurs précédents, l'analyse multivariée réalisée pour des raisons de validité statistique uniquement chez les femmes, a permis de faire ressortir chez les femmes hypertendues une hyponatrémie, une hypochlorémie, une hypo-protidémie, une hyperkaliémie, une hyperglycémie. Ces variations étaient aussi retrouvées significatives avec les analyses univariées. Si ces variations s'expliquent sans doute par le régime alimentaire hypo-salé et la prise de médicaments antihypertenseurs en majorité des diurétiques distaux et des bêtabloquants, l'hypoprotidémie dans ce contexte est d'explication plus difficile et mériterait d'être plus explorée. Le fort dimorphisme sexuel de l'HTA observée constitue une autre importante caractéristique de notre étude. La physiopathologie de l'HTA présente des spécificités liées au sexe [43]. De plus, nous avons observé une plus forte prévalence du diabète chez les femmes hypertendues que chez les hommes. Cela pourrait expliquer la plus forte proportion de paramètres biochimiques modifiés chez les femmes et l'obtention d'un modèle multivarié que nous n'avons pu obtenir chez les hommes. Enfin, les habitudes culturelles pourraient aussi expliquer ce dimorphisme, en terme d'adhésion au traitement ou au régime hyposodé, ainsi que par la prise de traitements à base de plantes.

## Conclusion

Si des facteurs génétiques ont été mis en avant pour expliquer la précocité et la gravité de l'HTA chez les noirs africains, notre étude montre que les autres facteurs nutritionnels et environnementaux ont aussi un lien direct avec l'HTA comme la sédentarité, l'obésité, le diabète et hyperhomocystéinémie. Tous ces facteurs de risques sont potentiellement modifiables et devraient faire partie de la stratégie de prise en charge et de prévention de l'HTA en Afrique.

### Etat des connaissances actuelles sur le sujet

- C'est un problème de santé publique;
- Absence de marqueurs prédictifs de complications cardiovasculaires (CV).

### Contribution de notre étude à la connaissance

- L'hyperhomocystéinémie et l'augmentation du NT-ProBNP est très répandue dans la population hypertendue à Bamako.

## Conflits d’intérêts

Les auteurs ne déclarent aucun conflit d'intérêts.
